# The Role of the Proprietaries

**Published:** 1906-02

**Authors:** 


### The Role of the Proprietaries.

The attempt on the part of the American Medical Association to coerce certain manufacturers and certain medical journals by a process of boycott is in direct opposition to the principles of our republican form of business and personal liberty.

It is only quite recently that the Association has reached an apparent degree of numerical strength to warrant it in thus arbitrarily laying down the law to the manufacturer of proprietary remedies and to the prescribers and advertisers of same, and the time has come for the individual physician to ask himself whether he will be the judge, and the only judge, of what he will prescribe or whether he will surrender his rights and ask the Association what he may be permitted to utilize in the treatment of disease! This is exactly the status today and it applies also equally to the independent journalist as to what he may and what he may not carry in his advertising pages.

That this process of intimidation and boycott has had its effect there can be no denial, for mushroom journals are springing into existence here and there as "State" journals, relying upon the support of those loyal to the Association and its methods; and there are certain manufacturers of proprietaries who have withdrawn their patronage from independent journals because they feared the influence of the Association. As we have said before, the whole thing at last rests with the rank and file of the profession, and as individuals it behooves us to look the field over and decide for ourselves the relation of the proprietors to the profession.

It must be admitted by the most prejudiced that the manufacturers of proprietary preparations have done more for the advancement of scientific therapeutics than any other class of men connected with the profession of medicine. The elegant and effective pharmaceuticals of today contrasted with the cumbersome, crude, and nauseous remedies in vogue twenty-five or thirty years ago when we commenced the practice of medicine, bear testimony to the truth of the above statement, and the attempt on the part of the American Medical Association and its allies to cast opprobrium upon the manufacturers or their products because certain of them will not bend the knee to these self-constituted censors, is a step backward. This self-constituted authority to make or unmake a manufacturer, a physician or a medical journal by the simple edict of the Association's committee appointed for this purpose, will only work with a certain class who are either too timorous to stand up for principle or who are cunning enough to ride for awhile astraddle of the ethical fence.

These manufacturers are not in the business just for honor and sentiment, but for the money they expect to get out of their products, and for this very reason it is a guarantee that their products will be all that scientific knowledge can produce, for they know that their purity, efficacy, and scientific adaptability to the cure of disease will be put to the test by the prescriber, and the question can only be fairly settled by the physician in practice and he alone should decide upon the merits of the instruments in his hands—the A. M. A. and its hired allies to the contrary notwithstanding.

In all of the cross-firing between the manufacturer, the Committee on Pharmacy of the Association and the opposing independent journals, the question of ethics, pure and simple, is not the thing at issue, as it is made to appear. It is a question of dollars and cents, though speciously stated. It is a struggle to control the business of the manufacturing chemists and pharmacists, and keep them on their knees before this committee paying tribute for the privilege of being considered ethical and clean. It is an attempt to force every individual doctor in the land to drop his own independence and prescribe only such proprietaries as may be pronounced favorably upon by the Chicago heads. It is an outrageous attempt to influence the physician to withdraw his support from the independent journals and support the organs of the trust, and all for *filthy lucre*.

When a conscientious physician is called upon to treat a case of disease, it is his duty to avail himself of every means of curing that patient that his knowledge or experience brings to his aid, and if he finds his best remedies in unethical company, so-called, his duty is just the same. A remedy is used for its effect—not for its ethics; for its effect upon the patient, not for its rating among hired medical scribblers. The average medical man is not only ethical, pure, and gentlemanly, but in his daily work he has learned to be at least as good a judge of ethics and therapy as those who would instruct him for a purpose!—*The Southern Clinic.*

